# Changes in Self-reported Energy and Brain Volumes

**DOI:** 10.1093/geroni/igaa057.2833

**Published:** 2020-12-16

**Authors:** Qu Tian, Andrea Rosso, Nancy Glynn, Xiaonan Zhu, Caterina Rosano

**Affiliations:** 1 National Institute on Aging, Bethesda, Maryland, United States; 2 School of Public Health, University of Pittsburgh, Pittsburgh, Pennsylvania, United States

## Abstract

The brain demands and consumes more energy than any other organ. Lower perceived energy may indicate compromised brain health. Little empirical data exists on the association between perceived energy and brain structure. Neuroimaging was obtained in 300 participants (mean age=83±3 y/o, 40% blacks, 57%women) with repeated self-reported energy measures(scale0-10) in the past decade. Energy decline was computed as rate of change by linear mixed models(-0.06/year). Associations of energy decline with volumes of cognitive (dorsolateral prefrontal cortex, hippocampus) and motor (precentral gyrus, putamen, caudate) areas were examined using linear regression, adjusted for demographics and total gray matter atrophy. A steeper decline in energy was associated with smaller volumes of right putamen (p=0.013) and caudate (p=0.043), a trend in right precentral gyrus (p=0.085), but not in prefrontal cortex or hippocampus. Declining energy by self-report may indicate atrophy localized in subcortical motor areas. Studies to identify the mechanisms underlying these associations are warranted.

